# Kupffer cells abrogate homing and repopulation of allogeneic hepatic progenitors in injured liver site

**DOI:** 10.1186/s13287-024-03656-w

**Published:** 2024-02-20

**Authors:** Nasir Abbas, Kai You, Anteneh Getachew, Feima Wu, Muzammal Hussain, Xinping Huang, Yan Chen, Tingcai Pan, Yinxiong Li

**Affiliations:** 1 Center for Health Research, Guangdong Provincial Key Laboratory of Biocomputing, Guangzhou Institutes of Biomedicine and Health, Chinese Academy of Sciences, Guangzhou, 510530 China; 2 Present Address: Centre for Regenerative Medicine and Health (CRMH), Hong Kong Institute of Science and Innovation, Chinese Academy of Sciences, Hong Kong, Hong Kong SAR China; 3 Key Laboratory of Stem Cell and Regenerative Medicine, Guangzhou Institutes of Biomedicine and Health, Chinese Academy of Sciences, Guangzhou, 510530 China; 4 CAS Key Laboratory of Regenerative Biology, Guangzhou Institutes of Biomedicine and Health, Chinese Academy of Sciences, Guangzhou, 510530 China; 5 Department of Hepatobiliary Surgery II, Zhujiang Hospital, Southern Medical University, Guangzhou, 510280 Guangdong Province China; 6 State Key Laboratory of Respiratory Disease, Guangzhou, 510000 China; 7 China-New Zealand Joint Laboratory of Biomedicine and Health, Guangzhou, 510530 China; 8 Department of Biomedical Engineering, The University of Alabama at Birmingham, Birmingham, USA; 9 Department of Biochemistry and Molecular Pharmacology, New York University Grossman School of Medicine, New York, NY 10016 USA

**Keywords:** Liver injury, Liver repair, Stem cells homing, Kupffer cells, HSCs, Innate immune response

## Abstract

**Background:**

Allogeneic hepatocyte transplantation is an emerging approach to treat acute liver defects. However, durable engraftment of the transplanted cells remains a daunting task, as they are actively cleared by the recipient’s immune system. Therefore, a detailed understanding of the innate or adaptive immune cells-derived responses against allogeneic transplanted hepatic cells is the key to rationalize cell-based therapies.

**Methods:**

Here, we induced an acute inflammatory regenerative niche (3–96 h) on the surface of the liver by the application of cryo-injury (CI) to systematically evaluate the innate immune response against transplanted allogeneic hepatic progenitors in a sustained micro-inflammatory environment.

**Results:**

The resulting data highlighted that the injured site was significantly repopulated by alternating numbers of innate immune cells, including neutrophils, monocytes and Kupffer cells (KCs), from 3 to 96 h. The transplanted allo-HPs, engrafted 6 h post-injury, were collectively eliminated by the innate immune response within 24 h of transplantation. Selective depletion of the KCs demonstrated a delayed recruitment of monocytes from day 2 to day 6. In addition, the intrasplenic engraftment of the hepatic progenitors 54 h post-transplantation was dismantled by KCs, while a time-dependent better survival and translocation of the transplanted cells into the injured site could be observed in samples devoid of KCs.

**Conclusion:**

Overall, this study provides evidence that KCs ablation enables a better survival and integration of allo-HPs in a sustained liver inflammatory environment, having implications for rationalizing the cell-based therapeutic interventions against liver defects.

**Supplementary Information:**

The online version contains supplementary material available at 10.1186/s13287-024-03656-w.

## Background

A key pathological feature of the overwhelming liver damage is the massive mobilization of innate and adaptive immune responses leading to acute liver failure [1, 2]. Therapeutically, liver replacement, also known as orthotopic liver transplantation (OLT), remains an essential curative therapy for acute liver failure and end-stage liver injuries [3–5]. However, emerging data indicate that transplantation of healthy hepatocytes in acute and chronic liver failures may substitute the OLT in clinical practice [6–10].

Over the last decade, allogeneic hepatocyte transplantation has actively been pursued as an alternative approach to OLT for the cure of liver-based metabolic defects and acute liver failure [11, 12]. Although encouraging clinical benefits have been observed in patients receiving allogeneic hepatocyte transplantation [13, 14], durable engraftment of the transplanted cells despite using immunosuppression has not yet been achieved [13]. Generally, it is considered that a Kupffer cells (KCs)-derived pronounced hindrance is offered during the translocation of the transplanted cells from the portal spaces into the site of integration at the liver parenchyma, leading to their elimination [15–17]. It has also been reported that the exogenous transplanted cells are recognized by activated instant blood-mediated inflammatory reaction [18–22], in combination with cytokines and chemokines primarily derived from neutrophils and KCs [15, 23]. Recent experimental evidence suggests that survival of the cellular allograft is limited by multiple barriers, including the endothelial lining of the sinusoids and immune rejection followed by spontaneous apoptosis [24–28].

A general drawback associated with cell-based therapies is the limited capacity of transplanted cells to repopulate, as of being unable to proliferate, in the normal liver and, thus, are actively cleared by the recipient immune system [28, 29]. For instance, a previous study by Joseph et al. [15] claimed that almost 70–80% of the transplanted syngeneic hepatocytes were immediately eliminated by the initial innate immune response, while it posed a significant hurdle for the remaining ones to be located and screened for biological activity and repopulation efficiency in the recipient's liver [30]. Given this fact, whether the innate immune cells, particularly the KCs, respond against allogeneic transplanted hepatic progenitors (allo-HPs), in the context of sustained liver inflammation, is not yet clearly defined.

Herein, by recapitulating the previously established protocol [31], we have generated allogeneic hepatic progenitors (allo-HPs) from adult hepatocytes isolated from BALB/c mice. These progenitors were then intrasplenically engrafted into C57BL/6 wild-type mice while inducing selective damage to their hepatic parenchyma. Our results indicate that a deleterious initial blood-mediated innate immune response, mainly governed by KCs, is instigated against the transplanted allo-HPs. By in vivo depletion of neutrophils and KCs, we further demonstrate that KCs ablation supports the viability of the allo-HPs that later translocated from portal spaces and repopulated at the injured site along with the hepatic stellate cells (HSCs). In brief, the findings reported in this study could be helpful for the rational optimization of transient cell-based therapies being evaluated in the context of sustained innate inflammatory response.

## Methods

### Ethical statement

Animal procedures were performed in the animal house facility of Guangzhou Institutes of Biomedicine and Health (GIBH), Chinese Academy of Sciences (CAS). All experimental procedures were approved by the ethical committee of GIBH (ethical process number: N2014050) and performed under the guidelines of the Guangdong Provincial Department of Science and Technology for Animal Welfare. Further, the animal studies are reported in compliance with ARRIVE guidelines. All efforts were made to ensure minimal animal suffering.

### Animal source

Female C57BL/6 and BALB/c mice, six to eight weeks old were purchased from Vital River Laboratory Animal Technology Co. (Beijing). For in vivo experiments, 90 mice were randomized into 30 experimental and control groups with 3 mice each (*n* = 3) The sample size was determined based on our and previous studies [32, 33]. All mice (4–5 per cage) were housed in a 12-h light/12-h dark cycle and were raised in pathogen-free grade cages in a temperature-controlled, sterile animal facility with free access to food and water.

### Induction of cryo-injury

For in vivo experiments, cryo-injury (CI) procedure was conducted as previously described [34]. Briefly, a midline laparotomy was performed under tribromoethanol (TBE) anesthesia in 0.9% of saline, and the left lobe of the liver was exposed. A metallic probe was immersed in liquid nitrogen and was carefully placed on the exposed lobe to achieve a 0.5 cm long lesion on the surface of the liver. The abdominal cavity was closed, and mice were recovered at 37 °C. Conversely, the animals in the sham/control group underwent the same procedure or sham injury (SI), except that the exposed liver was treated with a metallic probe set at room temperature. Animals were excluded from the study if any abnormal behavioral signs and/or ≥ 20% loss of body weight were observed. Mice were anesthetized by intraperitoneal (I.P) injection of TBE and were killed under specified intervals of time post-injury. In fact, the rationale behind selecting the Cryo-injury model for our study stems from the need to address limitations associated with conventional injury models (like BDL, CCl_4_, and APAP). These traditional models often induce widespread liver damage, which can impede the focused study of immune cell interactions with transplanted cells. In contrast, the Cryo-injury model creates a predetermined and marked injury loci, providing a unique feature for tracing the consequential events including the immune response and its impact on transplanted cells in a spatial–temporal manner.

### Assessment of cytokines by ELISA

Mice were anesthetized and blood samples were obtained by cardiac puncture and were collected either in a K3 EDTA vacutainer (cat# 367,861, BD) or in  an Eppendorf tube. Samples were centrifuged at 2000 RPM for 20 min and blood plasma was collected as supernatant, aliquoted and stored at—80 °C for downstream analyses. Standard procedures were followed according to the manufacturer’s instructions and blood cytokines levels were analyzed by standard ELISA kits for IFNγ (cat#: MIF00, R&D), IL-6 (cat #: M6000B) and IL-10 (cat#: BMS614-2). In addition, MCP-1 (Cat# AF-479, R&D) levels were analyzed by Western blotting.

### RNA isolation and gene expression analyses

Total RNA was extracted using TRIzol reagent (Invitrogen) from flash-frozen samples according to the manufacturer’s protocol. The isolated RNA was quantified (NanoDrop 2000—Thermos Fischer) was reverse transcribed into cDNA from 2 µg RNA using Revertra Ace (Toyobo) and oligo-dt (Takara). Quantitative RT-PCR was performed with the CFX96 and SYBR Green Premix system (Bio-Rad) following the manufacturer’s recommendations. The relative mRNA expression of the analyzed gene in all cases was normalized to β-actin, and each experiment was repeated thrice for validation. The primer sequences used in this study are mentioned Additional file 1: Table S1.

### ALT/AST assessment and histology

Serum levels of alanine aminotransferase (ALT) and aspartate transaminase (AST) were measured using a standard clinical automated analyzer (SRL, Tokyo, Japan). For histology, liver samples were harvested and washed with ice-cold PBS and were immediately fixed in 10% neutral buffered formalin for 24 h. Paraffin sections were stained with conventional hematoxylin and eosin (H&E) staining and examined.

### Immunohistochemistry and immunofluorescence

Mice were anesthetized and liver samples were collected and washed with ice-cold PBS. Harvested liver samples were either embedded in an optimum-cutting temperature (OCT) medium for cryostat section (7 µm) or fixed in 10% neutral buffered formalin 24 h for paraffin section (4 µm). Sections were incubated with 10% fetal bovine serum (FBS) for non-specific binding for 1 h at room temperature. Primary antibodies [anti-F4/80 (1:50; Bio-Rad: #MCA497, anti-CD11b (1:100; BD: # 550282), anti-CD45 (1:200; BD: # 550539), anti-Ly6G (1:150; Abcam: # ab25377), and αSMA (1:900; Abcam: # ab124964)] were diluted in 10% FBS and were incubated for 2 h at room temperature. Sections were washed with PBS and signals were amplified by secondary antibodies [ImmPRESS-HRP anti-rabbit IgG (Vector Labs # MP-7401) or ImmPRESS-AP anti-rat IgG (Vector Labs: # MP-5444)] for 40 min at room temperature. Immunogenic reaction was visualized by Alkaline Phosphatase (AP) Substrate (Vector Labs: # SK-5105), ImmPACT Vector Red Alkaline reagent (Vector Labs: # SK-5105) or DAB (Zsbio: # ZLI-9018) or with DAPI (1:2000) and mounted. All IHC images were quantified with ImageJ (NIH) in 5 random views at 40 × magnification as percentage of positive pixels in a blinded fashion.

### Induction of hepatic progenitors from primary mouse hepatocytes

Mouse HPs were generated as previously described [31]. Briefly, primary adult mice hepatocytes were isolated from 8 to 12 weeks old female BALB/c mice by two-step perfusion method. After perfusion with Ca^+2^ free Hank’s/EGTA solution and digestion with collagenase solution (Millipore Sigma), the digested livers were filtered and the suspension was collected via centrifugation at 50 g at 4 °C. Dead cells were removed, the remaining cells were washed twice and cultured with small hepatocytes basal medium (SHM) [DMEM/F12 (high glucose, Hyclone) supplemented with 5 mM HEPES (Sigma), 10 ng epidermal growth factor (PeproTech), 1% ITS (Gibco), 30 mg/L L-proline (Sigma), 0.05% BSA (Gibco), 10–7 M dexamethasone (Dex) (Selleck), 1 mM ascorbic acid, 10 mM nicotinamide (Stem Cell), and 1% penicillin and streptomycin solution (Life Technologies)] supplemented with 10% FBS (Gibco). Purified mouse hepatocytes were then seeded on collagen-coated plates in SHM with or without the combination of the small molecule inhibitors, Y-27632 (Selleck), 0.5 mM A-83–01 (Selleck) and CHIR99021 (Selleck). One day after seeding, the medium was changed and every other day thereafter. We confirmed all procedures for hepatic and biliary functions described for mouse hepatic progenitors as mentioned in [31].

### Luciferase reporter gene assay

Lenti-Combo Packing Mix (Cosmobio) and the expression construct pCDH-CMV-MCS-EF1-Greenpuro (System Biosciences, Mountain View, CA) were transfected into HEK293T cells to create lentiviral vectors (Clontech). The luciferase gene (GL3) (Promega) was inserted into the MCS of the expression construct. The culture medium was replaced with SHM + YAC a day after transfection. The clones were treated with collected lentiviruses two days later. Cells that had been transduced with GFP were chosen in SHM + YAC containing 2 g/mL puromycin (Life Technologies).

### Transplantation of allo-HPs

Wild-type C57BL/6 female mice were used as recipients for the hepatic transplantation of the allo-HPs. Liver was preconditioned with Clodronate liposomes (CLs) or PBS liposomes (PLs) before treatment(s). Acute liver damage was induced by CI in 6–8 weeks old mice 24 h post-PLs/CLs treatment(s). One to two days post-Cl, allo-HPs (1 × 10^6^) were intrasplenically transplanted into the recipient mice. For the experiments involving cell depletions, we established an internal experimental group. Animals under these groups underwent cryo-injury followed by transplantation of allogeneic hepatic progenitors (allo-HPs) but without any cell depletion inventions. These groups either receive phosphate-buffered saline liposomes (PLs) or isotype-Ly6G. In comparison, the external experimental groups, which were referred to as sham groups in our study, were established to provide a baseline reference for comparison. The animals in these groups underwent sham injury (SI) followed by allo-HPs without undergoing any cell depletion procedures. Samples were collected at specified time intervals, and GFP-tagged transplanted cells were monitored by confocal microscopy in liver samples. Further, to minimize the possibility of experimenter bias, the investigator who conducted the experiment was not aware of the treatment procedure.

### Kupffer cells and neutrophils depletion

CLs or PLs were prepared as previously described [35], and used for the depletion of KCs. Liposomes were brought to room temperature for 30 min before injection. The tail vein was sterilized with 75% ethanol. Intravenous injections of the CLs or PLs, according to the manufacturer’s instructions, were injected into 6–8 weeks old female C57BL/6 mice 24 h before inducing CI. Mice were killed under anesthesia and samples were collected after a specified interval of time. KCs depletion was confirmed by immunohistochemical staining of the sections with KCs specific marker F4/80. Considering the observed poor expression of CLEC4F on Kupffer cells in various models of liver inflammation and injuries, such as NASH [36–38] and LPS [39], coupled with the documented loss of CLEC4F expression in primary Kupffer cell cultures and the complete absence of CLEC4F expression in the Kupffer cell line [40, 41], we have chosen to utilize the F4/80 marker. F4/80 is recognized as the most widely used marker for Kupffer cells, as supported by landmark studies [42, 43]. The ultimate aim was to minimize the risk of misidentifying Kupffer cells in the context of our injury model.

Neutrophils were depleted 24 h before CI by a single intraperitoneal injection of 400 µg of anti-Ly6G (clone 1A8, BioXCell), as previously described [44]. To reduce the experimenter bias, the researcher was blind to the treatment procedure. Mice were killed under anesthesia and liver samples were collected as specified. Neutrophils depletion was confirmed by IHC staining of anti-Ly6G (Cat# 25377, 1:150, Abcam), a granulocyte-specific marker.

### Western blotting

Western blot was performed as previously described [45]. Briefly, sodium dodecyl sulfate–polyacrylamide gel electrophoresis (SDS-PAGE) of the extracted proteins from the injured site was subsequently blotted using anti-CCL2/MCP1 (Cat# AF-479, R&D) and  anti-GAPDH (Cat # 600041-I-Ig, Proteintech) antibodies.

### Statistical analyses

Statistical analysis was conducted using GraphPad Prism 7.0 software (GraphPad Prism Software Inc., San Diego, CA, USA). To determine the significance of differences between groups, Student's t-test or one-way analysis of variance (ANOVA) was employed. Statistical significance was denoted by *, **, ***, and ****, indicating *P*-values less than 0.05, 0.01, 0.001, and 0.0001, respectively. The data are presented as mean ± standard deviation. Representative results from three independent experiments are displayed.

## Results

### Characteristics of CI-induced hepatic inflammatory-and-regenerative niche

**Fig. 1:**
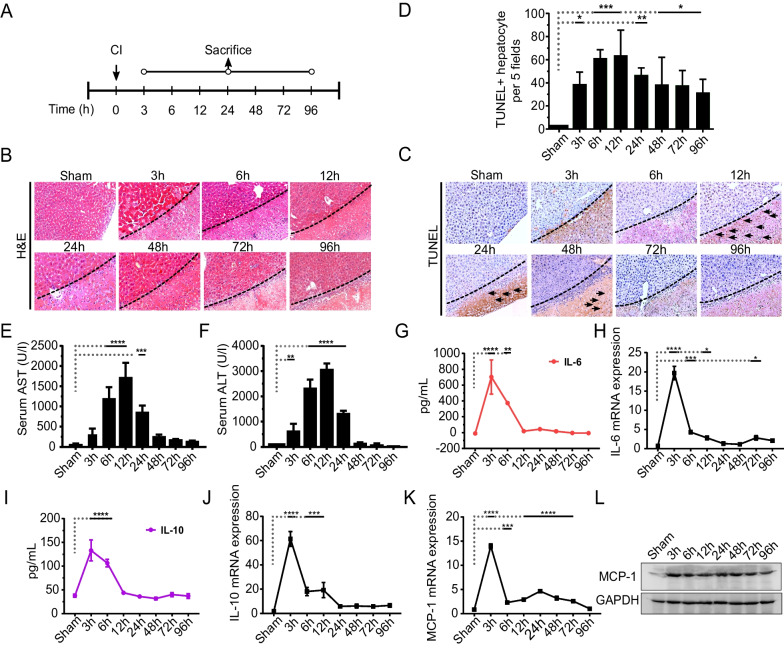
Generation of a regenerative niche by cryo-injury (CI). **A** The experimental design of induction of CI and sample collection at various time intervals is shown. **B** Representative H&E staining images of the sham and samples after CI. The dotted line indicates the boundary of the healthy and injured liver tissue. **C** Representative images from TUNEL assay, arrows indicate random necrotic hepatocytes after CI. **D** TUNEL staining was quantified by counting apoptotic hepatic nuclei in 5 random views at 40 × magnification as shown at indicated time intervals. Data are presented as mean ± SD, *p < 0.05, **p < 0.01 ***p < 0.001, *n* = 3/24 for each time point, scale bars: 100 µm. **E**, **F** Serum aspartate aminotransferase (AST) and alanine aminotransferase (ALT) analyses from mice with or without CI. **G**, **H** Interleukin-6 (IL-6) ELISA and IL-6 mRNA gene expression from the injured and non-injured liver is represented. **I**, **J** Interleukin-10 (IL-10) ELISA and IL-10 mRNA gene expression levels are shown. **K**, **L** Monocyte chemoattractant protein—1(MCP-1) mRNA gene expression and representative cropped immunoblot analyses from injured liver tissues, respectively. The corresponding full length immunoblot can be found in supplementary figure S4. Data represented as mean ± SD, *p < 0.05, **p < 0.01 ***p < 0.001, ****p < 0.0001., *n* = 3/24 per time point

We used our previously reported CI protocol [34] to establish a localized niche as a regenerative stimulus for the subsequent homing and repopulation of the transplanted hepatic progenitors. Figure 1A shows the experimental design and sample(s) collection at various time intervals after induction of cryo-injury. We detected a rapid onset of localized hepatic tissue damage, as characterized by conventional H&E stain (Fig. 1B) and TUNEL assay (Fig. 1C). The maximum induction of histological necrosis was significantly observed after 12 h of CI-induced hepatic damage (Fig. 1D). At the molecular level, the plasma levels of AST and ALT increased sharply and reached their peak level at 12 h after CI which corresponded with the progressed necrosis and tissue damage (Fig. 1E and F). In line with this, the serum levels and gene expression pattern(s) for both pro- and anti-inflammatory cytokines, such as interleukin (IL)-6 and IL-10 respectively, were significantly elevated at early 3 h, although a dramatic decline was observed thereafter (Fig. 1G–J). The monocyte chemoattractant protein-1 (MCP-1) also demonstrated a similar trend at gene expression level (Fig. 1K). However, we observed a consistent expression of MCP-1 at protein level, as indicated by Western blot analysis of samples prepared from liver tissue lysate after CI (Fig. 1L). Collectively, these results suggested that the CI induced the localized regenerative niche in the settings of acute inflammatory response.

### Granulocytes and monocytes selectively repopulate at the injured site

**Fig. 2:**
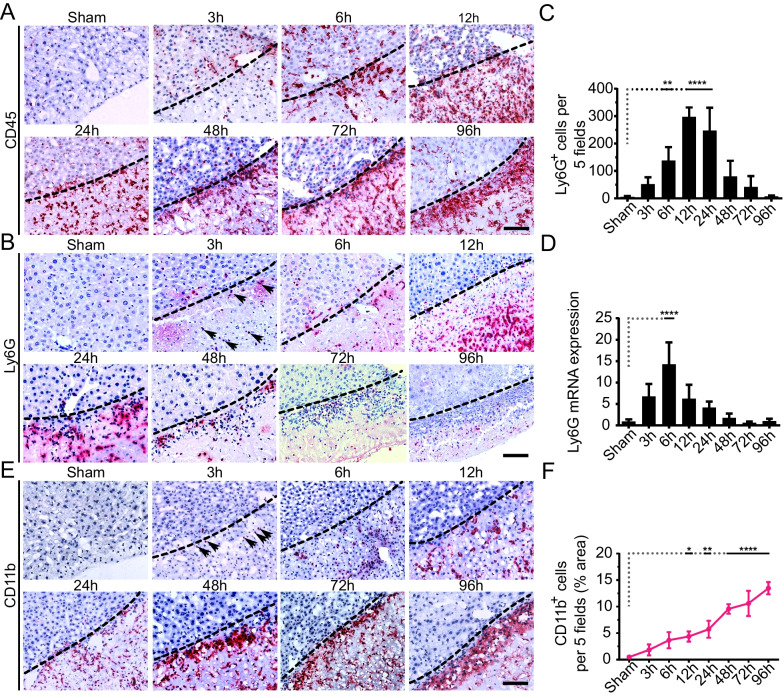
The sequential recruitment of neutrophils and monocytes into the injured site post-CI. **A** Representative images for pan-CD45 + (leukocytes) cells at specified time points post-CI. The dotted line represents the boundary of the injured site. **B** Representative IHC staining images of Ly6G + cells at the indicated time intervals. **C** Quantitation of Ly6G^+^ cells fields are shown. Ly6G^+^ cells were quantified in 5 random fields by quantifying positive cells per sample, *n* = 3/24 per time point. The data were averaged and presented as mean ± SD. **P < 0.01, ****p < 0.0001. **D** mRNA expression of Ly6G at the injured liver as compared to sham control. **E** Representative images for CD11b^+^ immunostaining showing monocyte trafficking at the injured site at the indicated time intervals. **F** Quantitation of CD11b^+^ cells per 5 fields is shown. CD11b^+^ cells are quantified by quantifying positive pixels in 5 random fields, averaged, and presented as mean ± SD of the positive area. *n* = 3/24 per time point. *p < 0.05, **p < 0.01, ****p < 0.0001. Scale bars 100 µm. Arrows (in **B** and **E**) indicate the infiltration of neutrophils (Ly6G^+^) or monocytes (CD11b^+^) at 3 h

The timely recruitment of blood-derived immune cells, such as neutrophils and monocytes, is the key feature of inflammatory response [46, 47]. Consistent expression of MCP-1 (Fig. 1L), which is a primary mediator of immune cells recruitment at the injured site [48], prompted us to enquire whether there is the infiltration of blood-derived granulocytes and monocytes at the injured site. To examine this, we performed an IHC analysis of liver tissue samples with a pan-CD45 marker. The results indicated that the CD45 + leukocytes began to infiltrate the injured site within 3-6 h, subsequently occupying the injured site from 12 to 24 h, and then gradually enriched at the proximity of the injured border (Fig. 2A). We next conducted an IHC analysis to determine the infiltration of neutrophils and monocytes into the injured site. Interestingly, neutrophil (Ly6G +) swarms were significantly detected into the injured site from 12 to 24 h post-CI (Fig. 2B, C). However, the overall neutrophil infiltration subsided soon after 24 h and only few cells could be observed after 48 h post-CI as compared to sham control (Fig. 2B, C). Likewise, a significantly upregulated mRNA expression of Ly6G was detected at 6 h, which gradually declined at the subsequent time intervals (Fig. 2D). In contrast, CD11b staining revealed a gradual, but consistent, infiltration of monocytes from 12 to 96 h (Fig. 2E, F). Overall, there was a parallel infiltration of the neutrophils (Ly6G +) and monocytes (CD11b +) from 3 to 24 h. Nevertheless, in the later time intervals, the patterns of infiltration altered conversely, with a consistent increase in the number of monocytes while a gradual decrease in neutrophils. It demonstrated that the CI recruited neutrophils and monocytes in a sequential manner.

### KCs accumulation induce the onset of regenerative responses at the injured site

**Fig. 3:**
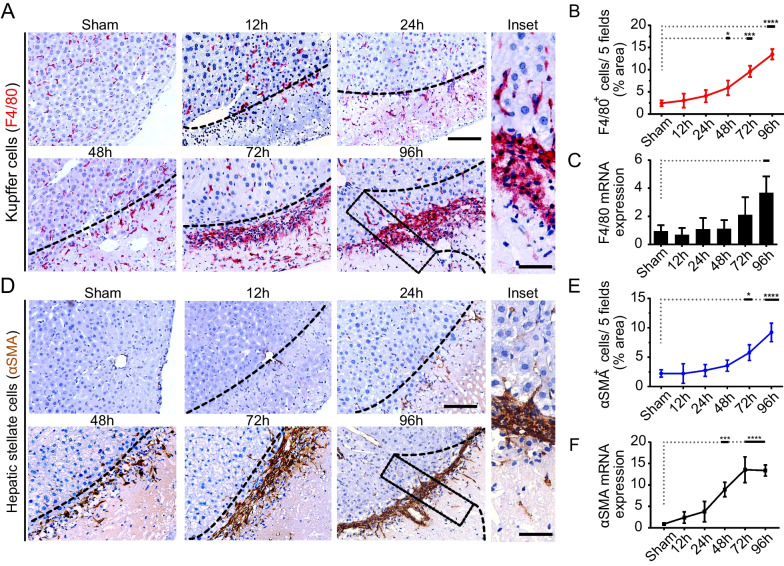
KCs accumulation at the injured site induces recruitment of HSCs mediating liver repair response. **A** Representative IHC images for F4/80^+^ cells (KCs) at indicated time intervals. Scale bars 100 µm (inset = 50 µm). **B**, **C** Quantitation of IHC images of F4/80^+^ cells, and F4/80 mRNA gene expression, respectively. F4/80 + cells are quantified by quantifying positive pixels in 5 random fields, averaged, and presented as mean ± SD. *n* = 3/24 per time point. Scale bars 100 µm, *n* = 3/24 per group. *P < 0.05, ***p < 0.001, and ****p < 0.0001. **D** Representative IHC images for αSMA expression indicating preliminary activation of HSCs at 24 h and subsequent time intervals. Scale bars 100 µm (inset = 50 µm). **E**, **F** Quantitation of αSMA^+^ cells (quantified by quantifying positive pixels in 5 random fields, averaged, and presented as mean ± SD. *n* = 3/24 per time point. Scale bars 100 µm, *n* = 3/24 per group. *P < 0.05, and ****p < 0.0001) and αSMA mRNA gene expression at indicated time intervals, respectively. αSMA mRNA data represented as mean ± SD, ***p < 0.001, and ****p < 0.0001., *n* = 3/24 per group

Emerging evidence indicate that liver parenchymal damage may induce inflammation and repair responses by the activation of KCs [49, 50]. As early as 12 h post-CI, the response timeline analysis for injury indicated that hepatic resident KCs (F4/80 +) started to accumulate near the injured site and seemed to migrate toward and through the injured border line at 48 h post-CI. Moreover, the accumulation of KCs in the injured site was progressively increased from 48 h to 96 h and formed an intensive border fence that separate the injured and non-injured tissue (Fig. 3A, B, inset). Likewise, the elevated gene expression of F4/80 was also detected in a time-dependent manner (Fig. 3C). Since wound repair is the manifestation of trans-differentiated HSCs into collagen-producing myofibroblasts after an injury [51, 52], the αSMA IHC analysis revealed active HSCs appeared in injured site at 24 h post-injury, it was 12 h later compared to appearance of KCs (Fig. 3D, E). However, the migration pattern was in accordance with the accumulation of KCs in later time intervals, but formed a tight border fence. Furthermore, the αSMA gene expression followed a similar trend as was observed with F4/80 at the indicated time intervals (Fig. 3F). Together these results suggest that regenerative responses are preceded by KCs, and the migration and accumulation pattern were followed by the activated HSCs in 12 h later.

### Depletion of KCs significantly reduces the recruitment of monocytes into the injured site

**Fig. 4:**
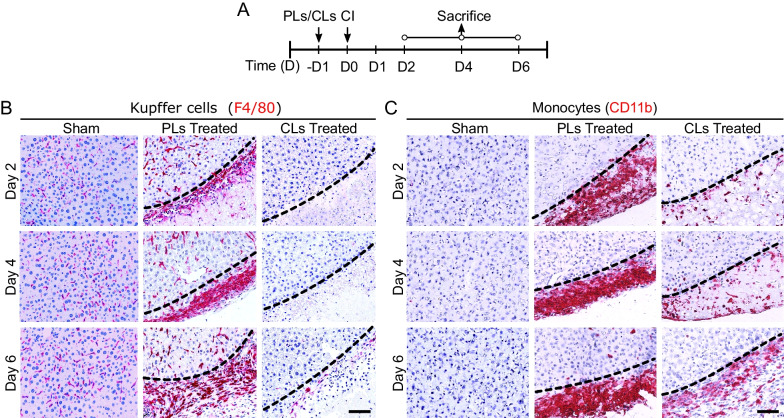
Depletion of Kupffer cells (KCs) substantially reduces the recruitment of monocytes from blood. **A** Experimental design of intravenous injection of either PBS liposomes (PLs) or Clodronate liposomes (CLs), induction of CI procedures and samples collection at specified time intervals. **B** Representative images of depletion of KCs shown in CLs-treated samples in comparison to PLs-treated and sham sections. KCs after depletion began to reappear at the injury site at day 6 post-CI. **C** Effect of KCs depletion on monocytes in PLs/CLs-treated samples as compared to sham samples. CLs treatment delayed the recruitment of monocytes, scale bar 100 µm, *n* = 3/27 per time point

Monocytes can rapidly extravasate into the inflamed tissue [47], and may conditionally repopulate to regain the lost pool of KCs [53]. To investigate whether modulating KCs activity may affect recruitment and repopulation of the monocytes, we administered 200 μl of either clodronate liposomes (CLs, the experimental group) or PBS liposomes (PLs, the control group) to C57BL/6 WT mice through tail vein in order to eliminate the KCs in liver. The experimental design and sample(s) collection at different time intervals are shown in Fig. 4A. Immunostaining displayed a time-dependent effect of PLs and CLs treatments on the repopulation of KCs at the injured site. Especially, there was a noteworthy accumulation of KCs in case of PLs-treated samples, which gradually increased from day 2 to day 6 (Fig. 4B). However, in the CLs-treated samples, those KCs were absolutely absent from day 2 to day 4 (Fig. 4B), although they emerged as a thin disconnected line in day 6 (Fig. 4B). To further elaborate whether the infiltration and subsequent repopulation of monocytes is dependent on KCs, we detected the localization of monocytes at the injured site by CD11b immunostaining of treated and untreated samples. Overall, a similar pattern of recruitment of monocytes was significantly decreased in day 2—day 6, while the KCs were depleted (Fig. 4C), even though there was a considerable accumulation of monocytes in day 6 of CLs-treated samples, but far less compared to the PLs controls. It indicated that the recruitment and the differentiation of monocytes as KCs are mainly dependent on the recruitment of primary KCs in liver.

### KCs hindrance in sinusoid affects the survival of transplanted allo-HPs

**Fig. 5:**
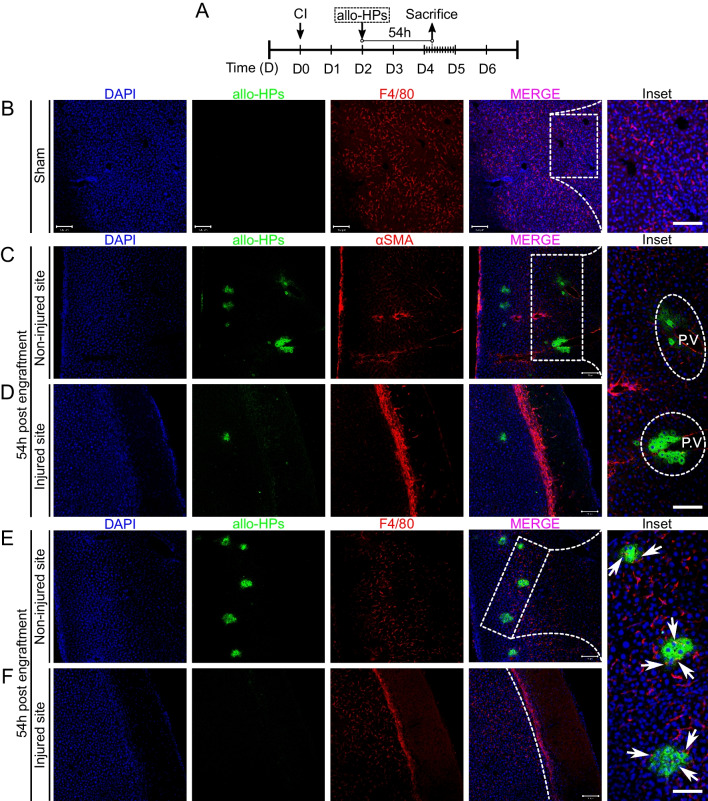
KCs hindrance affects the translocation of allo-HPs in the sinusoids. **A** Experimental design: CI was induced at day 0 (D0), and GFP tag allo-HPs were engrafted 48 h after induction of CI in wild-type C57 mice. Samples were subsequently collected 54 h post-engraftment. **B** Representative images of sham-treated liver samples post-transplantation are shown. Scale bars 100 µm (inset = 50 µm). **C** Transplanted allo-HPs were localized in the portal vein (PV) at the non-injured site. Scale bars 100 µm (inset = 50 µm). **D** Very few allo-HPs were detected at the vicinity of the injured site. **E** KCs dismantle allo-HPs in the sinusoids (inset = 50 µm, non-injured site). The arrows indicate KCs invading the clusters of allo-HPs. **F** Injured site representing KCs (F4/80^+^) cells. Scale bar 100 µm (inset = 50 µm), *n* = 3/27

Optimal engraftment of transplanted cells yet remains a daunting task [28]. The critical factors associated with the failure of the engraftment of the transplanted cells are mainly attributed to the sinusoidal effects, oxidative stress, and cytokine-mediated toxicity [28]. For instance, a previous study has shown that more than 70% of syngeneic transplanted hepatocytes are eliminated by preliminary innate immune response occurring within 24–48 h in the intact liver. However, being an integral component of the innate immune system, the response of KCs against miss-matched transplanted cells in the inflamed liver is still obscure [28, 29]. To do a preliminary assessment, we determined their post-transplantation hindrance effect on the translocation of allo-HPs from the portal vein to the injured site. We selected similar time points for the transplantation when KCs initiated to accumulate into the injured site (as mentioned in Fig. 3A). The experimental design and time intervals of samples collection are shown in Fig. 5A. The GFP histochemistry analysis at 54 h post-transplantation revealed that allo-HPs can readily be observed in portal areas and periportal spaces as compared to sham samples as mentioned in Fig. 5B, C. Most of the transplanted cells were entrapped in portal areas (enclosed in circles), and periportal liver sinusoids in non-injured site (Fig. 5C, inset). In addition, a cluster of transplanted allo-HPs translocated from portal areas was found to be localized at the vicinity of the invasive edge of the injured site as compared to sham controls (Fig. 5D). We also found that clusters of transplanted allo-HPs were entrapped in the liver plates at the distal end of the healthy site (Fig. 5E). Furthermore, the entrapped transplanted allo-HPs clusters were surrounded by phagocytes/macrophages (F4/80 + cells), which caused diffused fluorescence of the GFP tag and further suggested the preparatory dismantling activity of KCs (Fig. 5E-inset, marked by white arrows). Collectively, these results suggest that KCs hindrance in sinusoids affects the translocation of transplanted allo-HPs in the liver vasculature.

### KCs ablation and HSCs accumulation support survival and homing of transplanted allo-HPs in the injured site

We next sought to determine whether depletion of KCs affected the fate of the transplanted cells. Based on aforementioned results (in Fig. 5), we hypothesized that KCs might affect the survival of allo-HPs which, in turn, may reduce their homing and subsequent integration into the injured site. To test this, we first partially depleted KCs by intravenous injection of CLs 24 h before induction of CI (Additional file 1: Fig. S1). Allo-HPs were transplanted 48 h after CI and samples were collected post-54 h transplantation (Additional file 1: Fig. S1A). Interestingly, the dual F4/80^+^ and GFP histochemistry analyses showed that there was no accumulation of translocated allo-HPs into the injured site of the PLs-treated section at 54 h post-transplantation (Additional file 1: Fig. S1B). This probably owes to the expected hindrance effect imposed by KCs (F4/80 stained) that were observed to be linearly accumulated parallel to the injured site (Additional file 1: Fig. S1B), further evident by the detection of allo-HPs at the injured site in CLs-treated sections (Additional file 1: Fig. S1B, inset). The immunostaining analyses also showed that HSCs were considerably located in the CLs-treated samples (see Additional file 1: Fig. S1D, E). This, in addition to suggesting that the presence of HSCs (αSMA^+^) at the injured site is somehow affected by the presence or absence of KCs, hinted at their possible involvement in the homing of allo-HPs at the injured site.

Transplanted hepatocytes are either immediately cleared by innate immune cells [30], or lose their repopulation efficiency after engraftment [28]. Previous experimental evidence has established that transplanted hepatocytes sense beneficial signals from activated HSCs, which in turn can modulate their engraftment efficiency in rats [54, 55].

**Fig. 6:**
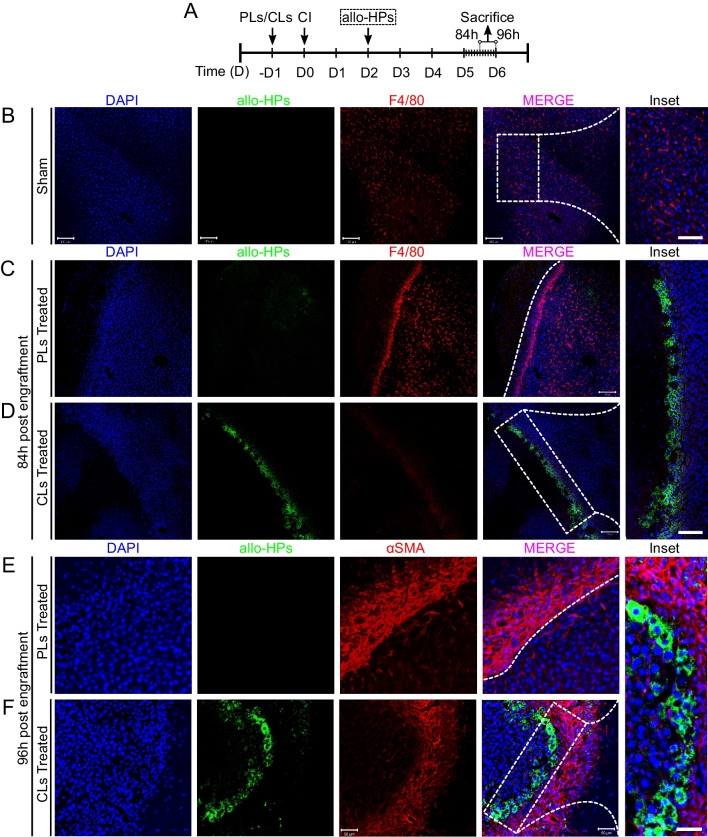
Engrafted GFP tag allo-HPs repopulating at the border of the injured site. **A** Experimental design: CI was induced at D0 and samples were collected 84 h or 96 h post-engraftment, respectively. **B** Representative images of sham-treated samples post-transplantation are shown. **C**, **D** PLs- and CLs-treated samples post-84 h of engraftment. CLs-treated samples show repopulation of allo-HPs at the border of injured site as compared to PLs-treated samples. **E**, **F** Representative images of engrafted allo-HPs post-96 h of engraftment. CLs-treated samples (inset = 20 µm) show repopulating allo-HPs at the border next to HSCs as compared to PLs-treated sections. Scale bar 100 µm, *n* = 3/27 per group

Considering this fact and our results from the initial assessment (in Additional file 1: Fig. S1), we questioned whether the accelerated kinetics of allo-HPs from portal spaces into the injured site is the manifestation of the inactivation of KCs with proactive support from the neighboring HSCs. To answer this, we increased the time interval from initial engraftment to sample collection (see Fig. 6A). To our interest, the analyses revealed that while there was a greater accumulation of the KCs 84 h post-transplantation in PLs-treated samples, an enriched prevalence of allo-HPs was observed at the injured site in CLs-treated ones (devoid of KCs) (compare Fig. 6B–D). The allo-HPs were particularly observed at the site of hepatocytes that were bordering the injured site. This hints  at either a paracrine or cell–cell support from the adjoining cellular source (possibly HSCs) that might retain allo-HPs at the injured site. To further elaborate on retention of allo-HPs at the injured site, we collected samples 96 h post-transplantation from PLs- and CLs-treated animals. Again, we found an increased accumulation of HSCs (αSMA^+^) in PLs sections as compared to CLs-treated sections (Fig. 6E). Of note, the PLs-treated sections remained devoid of traces of allo-HPs in the periphery and at the injured site. Since our transplanted cells were allogeneic, we were expecting a greater immune rejection despite KCs depletion. To our surprise, however, the CLs-treated sections showed a reasonable accumulation and integration of allo-HPs at the injured site (Fig. 6F). The allo-HPs and αSMA^+^ were observed to mutually adhered as adjoining cell types (Fig. 6F, inset), suggestive of mutual participation to enhance their integration at the injured site.

Overall, these data collectively suggest that the ablation of KCs supports initial survival and translocation of the allo-HPs into the liver injured site. Moreover, it also infers that allo-HPs repopulate in the vicinity of HSCs at the injured site, although the underlying mechanism(s) remains yet to be explored.

### Initial innate immune response collectively eliminates engrafted allo-HPs at early stages

The early innate immune response is critical for bio-distribution and acceptance of the initial cell graft post-transplantation [29, 56]. Previously, it was demonstrated that the invading granulocytes and leukocytes can infiltrate and kill the transplanted syngeneic hepatocytes [15]. Based on this fact, we next sought to investigate whether the survival of allo-HPs is affected by the initial innate immune response in the context of sustained inflammation after CI. For this purpose, we depleted KCs 24 h before induction of CI, and injected allo-HPs 6 h post-CI. Samples were collected 24 h post-transplantation (as shown in Additional file 1: Fig. S2A). The immunostaining (with CK19 marker for portal vein) revealed that while PLs-treated sections at 24 h post-transplantation were completely devoid of allo-HPs, the CLs-treated ones had a considerable number of allo-HPs mainly localized into the portal spaces (Additional file 1: Fig. S2B–D). Besides, the allo-HPs were observed to be protruding out from the CK19^+^ portal spaces (Additional file 1: Fig. S2E). These results suggested that the transplanted cells navigate the injured site through the portal vein.

**Fig. 7:**
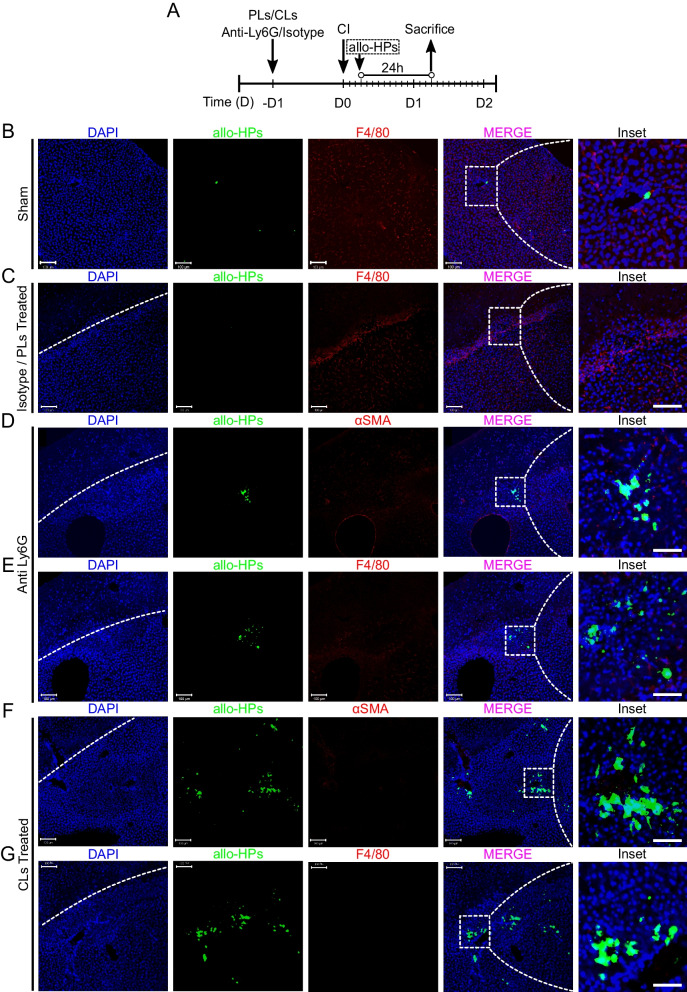
Initial innate immune response collectively eliminate engrafted allo-HPs at early stages. **A** Experimental design: anti-Ly6G and CLs/PLs were injected 24 h before CI, whereas allo-HPs were engrafted 6 h post-CI. The samples were collected post-24 h allo-HPs engraftment. **B** Representative images of sham-treated samples post-transplantation. **C** Isotype—or PLs-treated liver samples are presented. **D**, **E** Anti-Ly6G-treated samples representing few allo-HPs at the injured site. **F**, **G** CLs-treated samples representing substantially accumulated allo-HPs as compared to **D** and **E**. Scale bar 100 µm (inset = 50 µm), *n* = 3/27

Our above-described data in Fig. 2 indicated the presence of abundantly invading neutrophils into the injured site post-CI. To further explore whether neutrophils may affect the survival of allo-HPs post-transplantation, we carried out the depletion of neutrophils (by anti-Ly6G antibody) and KCs (PLs/CLs treatments) in two independent but parallel experiments. The experimental design of transplantation and samples collection is represented in Fig. 7A. The depletion of the neutrophils was confirmed by FACS analysis of the samples from peripheral blood (Additional file 1: Fig. S3). The F4/80^+^ and αSMA^+^ immunostaining of the sections treated with anti-Ly6G antibody highlighted an evident distribution of allo-HPs at the periphery of the portal spaces, as compared to absolute absence in case of isotype sections (Fig. 7B–E). To further clarify whether the existence of allo-HPs in the anti-Ly6g-treated samples is of the virtue of neutrophils, we analyzed CLs-treated sections by immunostaining of KCs (F4/80^+^) and HSCs (αSMA^+^). We found that allo-HPs were relatively more abundant and restricted to the portal spaces (in the form of clusters) as compared to isotype/PL and anti-Ly6G-treated sections (Fig. 7F, G, insets). The abundance of allo-HPs in CLs-treated samples, in comparison to anti-Ly6G ones, suggested the pronounced role of KCs in the elimination of transplanted cells (Fig. 7D–E). Altogether, these data suggest that initial innate immune response may collectively eliminate the transplanted allo-HPs in liver injury.

## Discussion

We previously demonstrated our strategy to protect transplanted liver progenitor cells driven from embryonic stem cells to escape the adoptive T cell response by ectopic expression of the CTLA4-Ig and PD-L1 molecules [57]. In this study, we mainly emphasize how innate immune cells, particularly the KCs, regulate the translocation and survival of the transplanted cells after a predefined liver injury.

We induced parenchymal liver damage to generate a niche to synergize liver inflammation and regeneration for the homing of the immune as well as transplanted cells; the model which is based on our previous report [34]. We first defined the timeline for the recruitment of the circulating myeloid cells (neutrophils and monocytes) and KCs, before the transplantation of the allo-HPs, to elaborate on how these cell types may modulate the survival of the transplanted cells. Our results implicate a potential coordinated activation of the post-injury cellular network characteristics of liver injury. For instance, given the integral features of acute inflammation [58], an initial transient upregulation in the levels of liver enzymes ALT/AST, as well as IL-6 and IL-10 cytokines, was considerably diminished within 12 h. Interestingly, despite parameters of acute liver injury being downregulated, we found a marked induction of MCP-1, which is a potent chemokine for the infiltration of the myeloid cells [48]. In turn, a rapid enrichment of the circulating myeloid cells (neutrophils/monocytes) was observed into the injured site after injury (Fig. 2). This is in accordance with a previous report where the fluctuating infiltration of these leucocyte subpopulations was demonstrated to be collectively participating and shifting the inflammatory microenvironment into the tissue remodeling niche [59].

The infiltration of the immune cells plays a key role in determining the viable outcome of wound repair. In the case of the injured liver tissue repair, it nevertheless particularly remains the concerted action of the KCs and HSCs [34]. We found both cell types were consistently enriched at the invasive edge of the injured site (Fig. 3A, D). Although not deeply explored here, the KCs-mediated recruitment of HSCs that we observed in this study seems to be an important factor in promoting wound healing (consistent with our previously reported findings in [34]). Besides HSCs, we also found that KCs depletion may essentially modulate the monocyte recruitment at the injured site (Fig. 4B, C), hinting that a possible cellular crosstalk between KCs and monocytes might exist post-injury. Indeed, monocytes and KCs have been reported to play an essential role during tissue repair [60]. Upon liver injury, KCs abundantly produce MCP-1 or CCL2 that especially recruit monocytes from the bone marrow as demonstrated in the choline-deficient, ethionine-supplemented diet model of liver injury and regeneration [60].

Extending our investigations to determine the fate of allo-HPs in the sinusoids, our data indicated that 72–96 h could be the most appropriate time slot for studying the potential impact of KCs (Fig. 3A, B) on transplanted cells. It is well acknowledged that the post-transplantation perturbation in the sinusoids temporarily occludes the blood flow which further aggravates the inflammation and liver injury by the activation of the KCs [30]. Animal studies have demonstrated that hepatocytes upon injection into the liver become trapped in sinusoids, leading to portal hypertension and ischemia–reperfusion injury [15]. The majority of infused hepatocytes remain trapped in the portal spaces and sinusoids, where they are eliminated by the innate immune system, including KCs and granulocytes [23]. In this context, the rapid mobilization of the KCs in the sinusoid and then colocalization with, and clearance of, the allo-HPs were not surprising (Fig. 5E). Interestingly, the elimination of the KCs demonstrated an accelerated translocation and integration of transplanted cells at the injured site (Fig. 6), which further substantiates the role of KCs in clearing transplanted cells in the sinusoidal areas. This appears to be in agreement with a previous report where the activation of KCs delayed the integration of the transplanted cells in the liver [16].

Our data further highlighted the post-transplantation events after KCs elimination. Indeed, one of the key obstacles for the transplanted cells is survival and proliferation in the host parenchyma [61], as engrafted cells incapable of integration are rapidly cleared from the sinusoids [30]. Notably, we observed that majority of transplanted cells that escaped the deleterious effects in the sinusoids were found to be concentrated and repopulated at the injured site (adjacent to the HSCs) in a time-dependent manner (Fig. 6F, inset). This, of course, is consistent with the previously reported findings where the HSCs were demonstrated to favor the engraftment of transplanted hepatocytes in the host liver [55, 62].

Moreover, this study advances our knowledge about the implication of the cumulative innate immune response before the allo-HPs are integrated into the liver parenchyma. A previous study has demonstrated that over 70% of the syngeneic transplanted cells are rejected due to sinusoidal effects [30]. In fact, allogeneic stem cells possess the least immune-privileged properties as compared to syngeneic cells [63]. They are mainly destroyed by the subsequent adaptive immune response once they are integrated into the liver architecture [64, 65]. However, both allogeneic and syngeneic transplanted liver cells may be targeted by the innate immune system in vitro [66, 67]. Consistently, our confocal studies demonstrated that neutrophils and KCs are the predominant effectors in the allogeneic cell rejection 24 h post-transplantation. Thus, the depletion of neutrophils and KCs essentially eliminated the pro-inflammatory response that is critical for the survival and homing of the transplanted cells (Fig. 7). This, in addition to further substantiating the role of neutrophils and KCs in the elimination of the allogeneic cells, provided a proof-of-concept validation for a previously established notion that the transplantation-induced inflammation is driven by neutrophils and KCs [23].

## Conclusion

This work clearly defines the role of neutrophils and KCs in the survival of allogeneic cells post-transplantation. In particular, we have shown that KCs depletion enables better translocation and homing of the transplanted cells at the injured site. Although the exact underlying mechanisms that determine that how the post-transplantation inflammatory microenvironment of the liver may impede cell engraftment remain obscure, the overall inflammatory responses demonstrated here as part of liver injury may aid in defining the organ-specific changes following cell transplantation and the subsequent cell-based therapy.

### Supplementary Information

**Additional file 1.** **Fig. S1.** KCs ablation supports survival of allo-HPs at the injury site. **Fig. S2.** Engrafted allo-HPs migrate and navigate to the injury site through portal vein (PV). **Fig. S3.** Depletion of neutrophils in peripheral blood. **Fig. S4.** Corresponding full-length immunoblot. **Supplementary Table S1:** List of primers for qPCR.

## Data Availability

The datasets supporting the conclusions of this article are included within the article.

## Abbreviations

CI: Cryo injury
KCs: Kupffer cells
allo-HPs: Allogeneic hepatic progenitors
CL: Clodronate liposomes
PL: PBS liposomes
OLT: Orthotopic liver transplantation
HSCs: Hepatic stellate cells
IL-6: Interleukin-6
IL-10: Interleukin-10
MCP-1: Monocyte chemoattractant protein-1
IFNγ: Interferon gamma
ALT: Alanine aminotransferase
AST: Aspartate transaminase
αSMA: Alpha smooth muscle actin
IHC: Immunohistochemistry
