# Supplementary material for: Kupffer cells abrogate homing and repopulation of allogeneic hepatic progenitors in injured liver site
Source: Stem Cell Res Ther. 2024 Feb 20;15:48. doi: 10.1186/s13287-024-03656-w (PMC10877762; doi:10.1186/s13287-024-03656-w)
Supplement: Supplementary file 1 — Additional file 1. Fig. S1. KCs ablation supports survival of allo-HPs at the injury site. Fig. S2. Engrafted allo-HPs migrate and navigate to the injury site through portal vein (PV). Fig. S3. Depletion of neutrophils in peripheral blood. Fig. S4. Corresponding full-length immunoblot. Supplementary Table S1: List of primers for qPCR. [file 13287_2024_3656_MOESM1_ESM.docx]

**Supporting information**

**Kupffer cells abrogate homing and repopulation of allogeneic hepatic progenitors in injured liver site**

**Nasir Abbas**^1,2^, **Kai You**^1^, **Anteneh Getachew**^8^, **Feima Wu**^1^, **Muzammal Hussain**^9^, **Xinping Huang**^1^, **Yan Chen**^1,3,4^, **Tingcai Pan**^5^, **Yinxiong Li**^1,3,4,6,7 *^

^1^ Center for Health Research, Guangdong Provincial Key Laboratory of Biocomputing, Guangzhou Institutes of Biomedicine and Health, Chinese Academy of Sciences, Guangzhou 510530, China

^2^ Current address: Centre for Regenerative Medicine and Health (CRMH), Hong Kong Institute of Science & Innovation, Chinese Academy of Sciences, Hong Kong SAR, China

^3^ Key Laboratory of Stem Cell and Regenerative Medicine, Guangzhou Institutes of Biomedicine and Health, Chinese Academy of Sciences, Guangzhou 510530, China

^4^ CAS Key Laboratory of Regenerative Biology, Guangzhou Institutes of Biomedicine and Health, Chinese Academy of Sciences, Guangzhou 510530, China

^5^ Department of Hepatobiliary Surgery II, Zhujiang Hospital, Southern Medical University, Guangzhou 510280, Guangdong Province, China

^6^ State Key Laboratory of Respiratory Disease, Guangzhou 510000, China

^7^ China-New Zealand Joint Laboratory of Biomedicine and Health, Guangzhou 510530, China

^8^ Department of Biomedical Engineering. The University of Alabama at Birmingham, USA

^9^ Department of Biochemistry and Molecular Pharmacology, New York University Grossman School of Medicine, New York, NY 10016, USA.

^*^ Correspondence: [li_yinxiong_iph@gibh.ac.cn](mailto:li_yinxiong_iph@gibh.ac.cn)

**
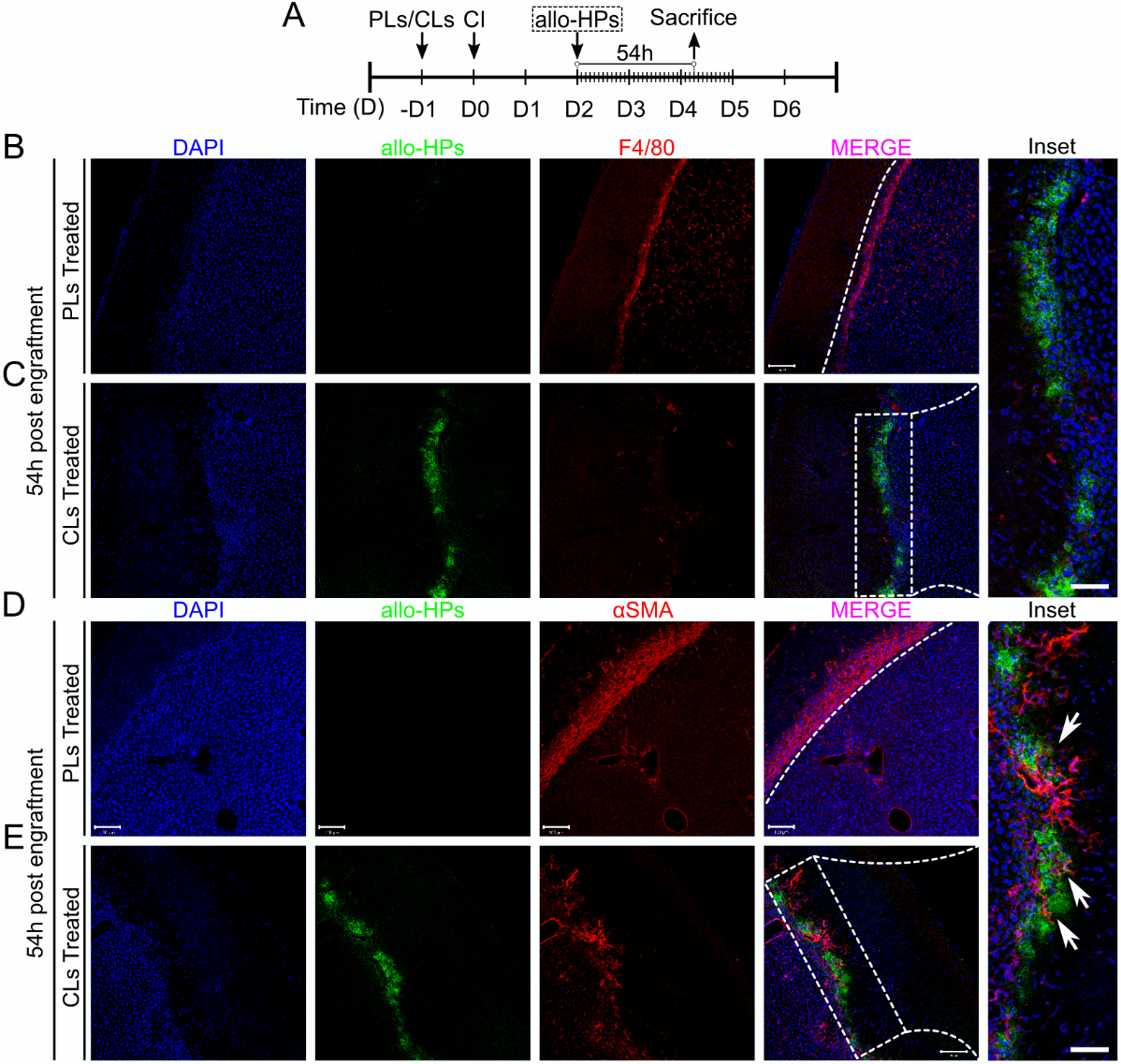
**

**Fig. S1. KCs ablation supports survival of allo-HPs at the injury site.** (A-B) Representative images of PLs- and CLs-treated samples. KCs-ablated samples show surviving allo-HPs while PLs-treated samples could not show any sign of engrafted allo-HPs. (C-D) Representative images showing engrafted allo-HPs localized with HSCs at the injury border in samples that were devoid of KCs. No surviving allo-HPs were observed in PLs-treated samples. Scale bar 100 µm (inset = 50 µm), n ≥ 3.


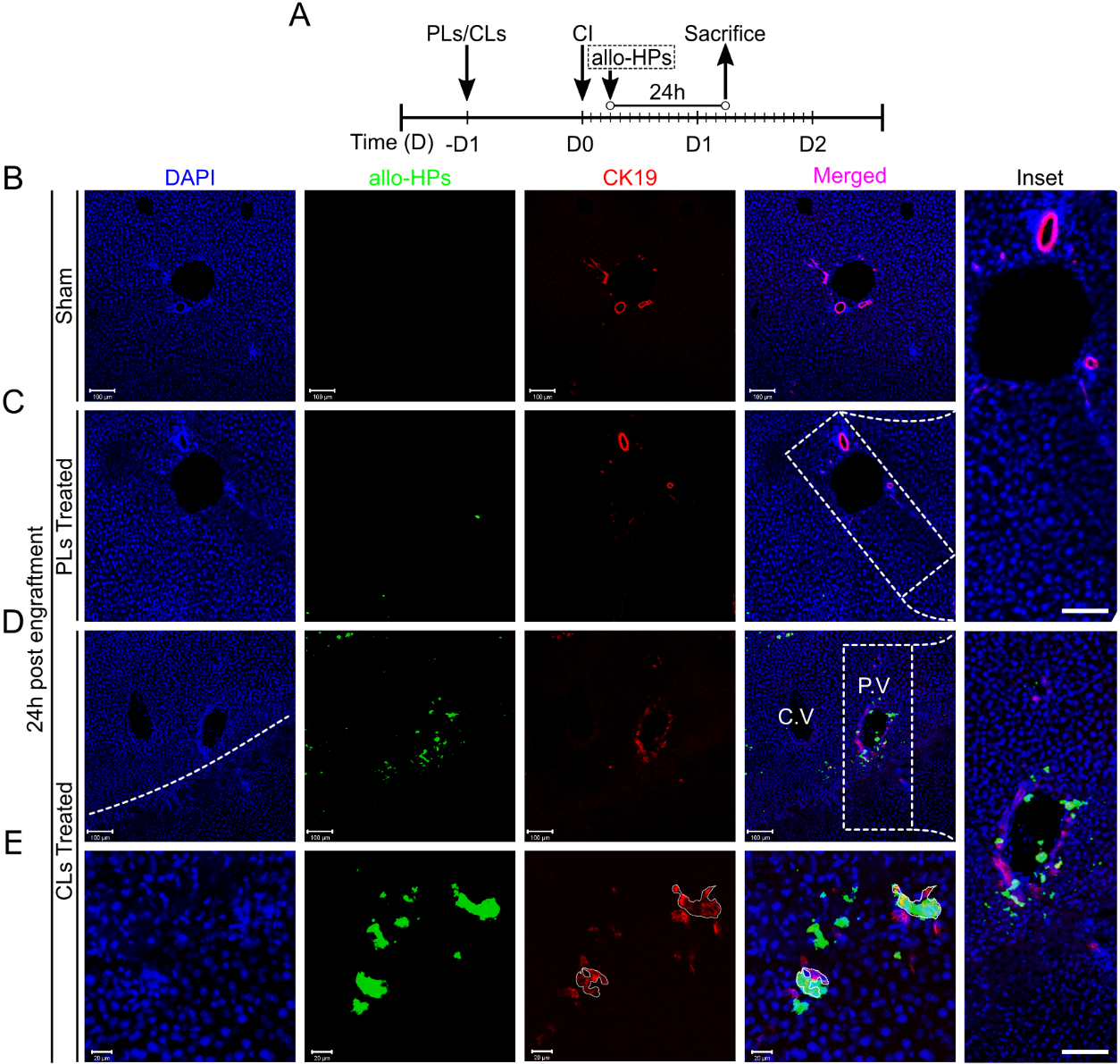


**Fig. S2 Engrafted allo-HPs migrate and navigate to the injury site through the portal vein (PV).** (A) Experimental design, PLs/CLs were injected 24h before injury, while allo-HPs were engrafted post-6h CI. (B) Sham treated samples. (C) PLs-treated samples with negligible allo-HPs near portal vein (inset). (D) Representative images of CLs-treated samples with CK19+ immunostaining. Allo-HPs can be seen surrounding the PV (inset). (E) Allo-HPs protruding out of CK19+ positive cells. The scale bar for B, C, and D is 100 µm (inset = 50 µm), Scale bar for E is 20 µm, n ≥ 3.


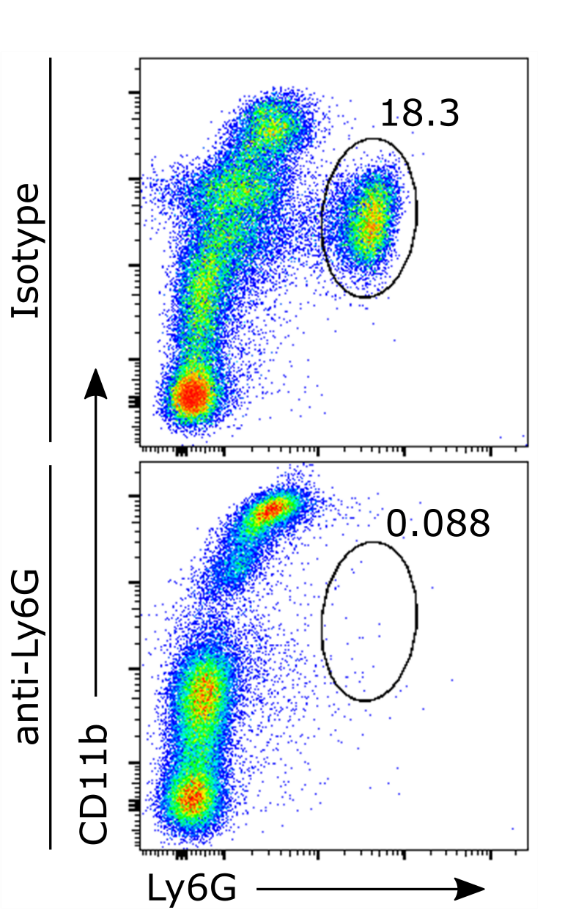


**Fig. S3 Depletion of neutrophils in peripheral blood.** Neutrophils were depleted *in vivo* by a single intraperitoneal injection of the anti-Ly6G antibody 24h after induction of CI. The efficacy of the depletion of the neutrophils is shown in FACs analysis which represents a marked reduction in the neutrophils as compared to isotype control in blood.


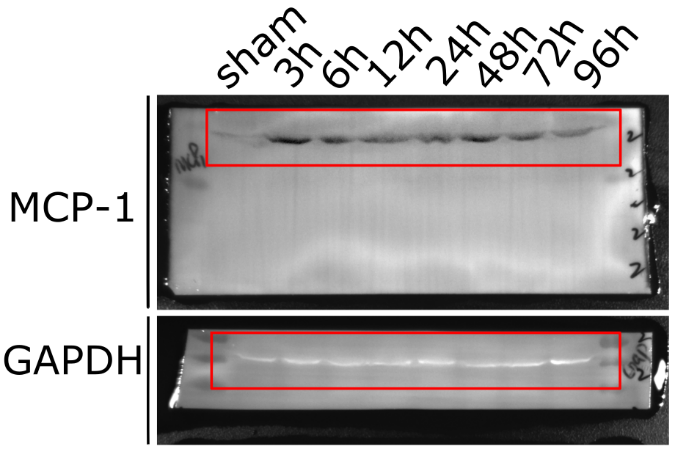


**Fig. S4 Corresponding full-length immunoblot:** Full-length immunoblot represents the MCP-1 and GAPDH. The red rectangles indicate the cropped immunoblot.

**Supplementary Table S1:** List of primers for qPCR

| **Gene** | **Primers Sequence** | |
| --- | --- | --- |
| β-actin primer | F | AGTGTGACGTTGACATCCGT |
|  | R | TTGCTGATCCACATCTGCTG |
| Ly6G | F | AGAGGAAGTTTTATCTGTGCAGCC |
|  | R | TCAGGTGGGACCCCAATACA |
| α-SMA | F | CCTGACTGAGCGTGGCTATT |
|  | R | AAGCGTTCGTTTCCAATGGTG |
| IL-6 | F | AGTTGCCTTCTTGGGACTGA |
|  | R | TCCACGATTTCCCAGAGAAC |
| F4/80 | F | CCCCAGTGTCCTTACAGAGTG |
|  | R | GTGCCCAGAGTGGATGTCT |
| MCP1 | F | AGGTCCCTGTCATGCTTCTG |
|  | R | TCTGGACCCATTCCTTCTTG |
| Mouse IL-10 | F | CTTACTGACTGGCATGAGGATCA |
|  | R | GCAGCTCTAGGAGCATGTGG |
